# Rapid Dilatation of the Uterine Canal for Dysmenorrhœa and Sterility

**Published:** 1885-05

**Authors:** 


### Selections.

### RAPID DILATATION OF THE UTERINE CANAL FOR DYSMENORRHŒA AND STERILITY.

At a recent meeting of the Philadelphia Obstetrical Society, Dr. Wm. Goodell brought forward his experience in rapid dilatation, and its advantages, as compared with the use of tents and Sims' cutting operation, both, formerly, his favorite modes of treatment.

He uses two sizes of Ellinger's dilators. He considers them best because their blades separate parallel to each other. The smaller is used to pilot the way for the more powerful one. The patient is fully anæsthetized and given a rectal suppository of the aqueous extract of opium. Dr. Goodell operates through his bivalve speculum, the patient on the back. The cervix is steadied by a strong tenaculum and the smaller dilator passed up as far as it will go. Upon gently stretching open that portion of the canal which it occupies, the stricture above so yields that when the instrument is closed it can be made to pass up higher. Thus by repetitions of this maneuvre, little by little, in a few minutes' time, a cervical canal is tunneled out which could not before admit the finest probe. Should the os externum be a mere pin-hole, or be too small to admit the beak of the dilator, it is enlarged by the closed blades of a straight pair of scissors, which are introduced with a turning motion. As soon as the cavity of the womb is gained, the handles are brought together, the smaller dilator being now withdrawn. The larger one is introduced, and the handles are then slowly screwed together. If the flexion be very marked, this instrument, after being withdrawn, should be retroduced with its curve reversed to that of the flexion, and the final dilation then made. The ether is now withheld, and the dilator kept in site until the patient begins to flinch." The best time for dilatation is midway between two monthly periods.

The patient is kept in bed till all pain and tenderness have passed away, rarely more than two or three suppositories are needed.

In his own words: "In the great majority of cases I dilate the canal, not to the fullest capacity of the instrument, but to one and a quarter inches. Sometimes an infantile cervix which does not readily yield and might give way, the handles are not screwed down more than three-quarters of an inch or an inch.

The cervical canal seldom returns to its contracted or angular condition. The cervix becomes shortened, widened, strengthened and straightened. He therefore uses rapid dilatation to straighten out anteflexed or retroflexed wombs, and to dilate and shorten the canal in cases of dysmenorrhœa or sterility from stenosis or from a cervical cervix. He also uses this means of dilatation to introduce tents, medicaments, the curette, to digitally explore the uterine cavity, and to irrigate the uterine cavity.

He has not a record of all cases so treated, but thinks they will number over three hundred. Out of these he has not had one case of severe inflammation, and the results have proved most satisfactory.

The following are the statistics for dysmenorrhœa:

| | |
|---|---|
| Unmarried, . . . . . . . . | 80 |
| Married, . . . . . . . . | 88 |
| Total, . . . . . . . . | 168 |

Of the unmarried eighteen were unheard from after the operation, leaving sixty-two from which any data could be obtained. Of these, thirty-eight were cured, seventeen more or less improved, and seven not improved at all. Of these seven, five subsequently had their ovaries removed. In each the ovaries had been so altered by cystic or by interstitial degeneration as to make the dysmenorrhœa otherwise incurable.

Of the married, fifty-three were heard from. Of these, thirty-nine were cured, ten improved, and four unimproved. And of these fifty-three, nine were not in condition to conceive, three of them from fibroid tumors, two from destructive applications of nitrate of silver to a lacerated cervix, three from being over forty-one years of age, and one a widow.

This leaves but forty-four capable of conception, and of these, eight, or a little over eighteen per cent., became pregnant. But the ratio is in fact larger, for several, fearing pregnancy, employed preventive measures after the operation. There have been many pregnancies occurring later, of which he has accidentally heard and which have not been reported to him by the patients. The dangers of the operation are of lighting up a former cellulitis or ovaritis. Dr. Goodell has not hesitated to operate, but always uses opium first, and by the time the operation is over, the patient is under its influence.—*American Journal of Obstetrics.*

